# Histological Tissue Response to Calcium Silicate-Based Cements Assessed in Human Tooth Culture Models: A Systematic Review

**DOI:** 10.3390/jfb17020078

**Published:** 2026-02-06

**Authors:** Alberto Cabrera-Fernández, Hebertt Gonzaga dos Santos Chaves, Aránzazu Díaz-Cuenca, Juan J. Segura-Egea, Jenifer Martín-González, João Peça, Diana B. Sequeira, João Miguel Marques dos Santos

**Affiliations:** 1 Department of Stomatology, Endodontic Section, School of Dentistry, University of Sevilla, 41009 Sevilla, Spain; acabrera5@us.es (A.C.-F.); segurajj@us.es (J.J.S.-E.); jmartin30@us.es (J.M.-G.); 2 Departament of Restorative Dentistry, Universidade Federal de Minas Gerais, Belo Horizonte 31270-901, MG, Brazil; heberttchaves_@hotmail.com; 3 Materials Science Institute of Seville (ICMS), Joint CSIC-University of Seville Center, 41092 Sevilla, Spain; aranzazu@icmse.csic.es; 4 Center for Neuroscience and Cell Biology, University of Coimbra, 3004-504 Coimbra, Portugal; jpeca@cnc.uc.pt (J.P.); disequeira@gmail.com (D.B.S.); 5 Institute of Endodontics, Faculty of Medicine, University of Coimbra, 3000-075 Coimbra, Portugal; 6 Center for Innovation and Research in Oral Sciences (CIROS), Faculty of Medicine, University of Coimbra, 3000-075 Coimbra, Portugal

**Keywords:** Biodentine, calcium silicate-based cements, dental pulp capping, human tooth culture model, reparative dentin, vital pulp therapy

## Abstract

Ex vivo human tooth culture models preserve the native dentine–pulp complex and offer a translational platform to study pulp-capping biomaterials. This systematic review aimed to synthesize the evidence on histological pulp tissue responses to calcium silicate-based cement (CSCs) used for direct pulp capping in human tooth culture models. The review followed PRISMA 2020 guidance. Eligible studies were ex vivo whole human tooth culture models with direct pulp exposure treated with commercial or experimental CSCs and reporting histological outcomes. Risk of bias was assessed using the QUIN tool. Thirteen studies were included. Most used immature human third molars (from 15- to 19-year-old patients) and culture periods up to 28 days, with a minority extending observation to 45–90 days. Across hydraulic CSCs, Biodentine was the most frequently evaluated material, followed by ProRoot MTA and several experimental hydraulic and resin-modified formulations. Overall, hydraulic CSCs were consistently associated with biocompatible pulp responses and a pro-mineralization pattern characterized by periexposure mineralized foci/osteodentin-like tissue; where assessed, immunohistochemistry supported odontoblast-like differentiation. In contrast, the resin-modified CSC TheraCal LC and other experimental resin-modified CSCs showed more heterogeneous findings, with reports of absent, delayed, or less prominent mineralization compared with reference hydraulic CSCs. In intact human tooth culture models, hydraulic CSCs show reproducible biocompatibility and early mineralization features consistent with reparative dentinogenesis, whereas resin-modified CSCs demonstrate more variable histological performance.

## 1. Introduction

Vital pulp therapy (VPT) is defined as the set of endodontic therapeutic procedures applied to teeth diagnosed with pulpitis, the aim of which is to control the inflammatory process and maintain pulp vitality, wholly or partially [1]. These procedures are grounded in the defensive and reparative potential of the dentine–pulp complex in response to injury [2]. Historically, VPT was mainly indicated for immature permanent teeth, where it achieved predictable success. More recently, advances in pulpal biology and the development of bioactive dental biomaterials have expanded its indications to mature permanent teeth with pulpitis, supporting the contemporary use of VPT in a broader range of clinical scenarios [3].

When a pulp exposure occurs, either due to a coronal fracture or during the removal of carious tissue, the insult is sufficiently intense to cause odontoblast death. An inflammatory process is then triggered, leading to the release of mediators and biochemical reactions that enable the migration and proliferation of multipotent mesenchymal stem cells present in the dental pulp, which will differentiate into cells similar to odontoblasts, known as ‘odontoblast-like cells’ [4]. This cell subpopulation is of great importance, as it secretes a mineralized matrix with mineral and molecular features comparable to dentine. The resulting mineralized tissue bridge protects the pulp from further injury and contributes to tissue healing and maintenance of vitality [5].

From a biological perspective, three requirements are necessary to achieve success in VPT procedures: (1) good systemic health and reparative capacity of the patient; (2) strict control of bacterial contamination; and (3) application of a bioactive material that stimulates the migration of pulpal stem cells to form reparative dentine [2].

Classically, the material used for this type of procedure has been Ca(OH)_2_. It is an alkaline material with strong antibacterial capacity, capable of solubilizing bioactive molecules, such as TGF-β1, involved in reparative dentinogenesis [6]. However, this material presents important drawbacks, including solubility, limited sealing capacity, undesirable irritation of the pulpal tissue, and the presence of tunnels and structural discontinuities in the resulting dentine bridge [7].

The introduction of calcium silicate-based cements (CSCs) represented a major shift in biomaterials used for vital pulp therapy (VPT). Torabinejad et al. (1993) first reported their use in endodontic applications with favourable in vitro outcomes [8], and the first widely adopted formulation was commercialized in 1998 as ProRoot MTA. Composed mainly of C_2_S and C_3_S phases, ProRoot MTA attracted substantial clinical and scientific interest due to its bioactivity, antibacterial effects, sealing ability and cellular biocompatibility [9]. However, it also presented relevant limitations including a long setting time, challenging handling, and the incorporation of Bi_2_O_3_ as a radiopacifier, which has been associated with tooth discoloration and cytotoxic effects at the cellular level [10].

In response to these shortcomings, CSCs have undergone rapid development, yielding multiple advanced formulations intended to overcome the limitations of ProRoot MTA [9,11], alongside an expansion of clinical indications that has consolidated CSCs as materials of choice for VPT procedures [12,13]. A pivotal milestone in this evolution was the launch of Biodentine, a second-generation tricalcium silicate-based cement introduced to provide improved handling and faster setting kinetics while maintaining the bioactive profile that underpinned MTA’s clinical success. Building on this trajectory, newer calcium silicate-based formulations have continued to prioritize operability and clinical versatility. Beyond conventional powder–liquid systems, ready-to-use premixed CSCs with putty-like consistency and accelerated setting reactions have been developed to facilitate manipulation in procedures such as pulpotomies and endodontic microsurgery, where precise placement and reduced technique sensitivity are desirable [14]. Furthermore, resin-modified calcium silicate materials have been designed to enhance placement accuracy and shorten setting time through light-activated polymerization, providing additional practical advantages during clinical application [15]. While these innovations may improve operability, formulation changes can also influence physicochemical behaviour and biological interactions with pulp tissue. Consequently, the growing diversity of calcium silicate-based biomaterials highlights the need for rigorous biological characterization to enable meaningful comparisons and support evidence-based clinical decision-making.

Throughout this evolutionary process, a large number of models have been published in endodontics to characterize the biological behaviour of hydraulic CSCs, ranging from in vitro models to in vivo models. In vitro models employ cells isolated from their natural environment to conduct assays outside the organism, under controlled laboratory conditions [16]. We find two-dimensional (2D) in vitro studies and 3D models that better represent cellular environments [17]. Although far from clinical conditions, numerous researchers have studied the biological behaviour of CSCs using these types of assays [18]. Animal experimentation represents another approach, classified as either ectopic or orthotopic models according to the anatomical site of the experimental procedure [17]. Orthotopic models implant the material in its natural location; for this purpose, larger animals are used, which implies higher cost and greater handling difficulty. In endodontic procedures, dogs and, likewise, porcine species are widely used because their dentitions show growth patterns and pathophysiology similar to human teeth [19,20,21]. Nevertheless, the pulpal response to biomaterials in animals may not fully replicate human tissue behaviour, which limits direct translation of findings [22].

To bridge the gap between simplified in vitro assays and animal experimentation, Téclès et al. (2008) proposed a culture model of an intact human tooth [23]. This is an ex vivo model in which a whole organ, recently extracted and with minimal modification of its natural state, preserves vitality under controlled laboratory conditions so that the cells remain viable. This methodology has the major advantage of assessing cellular and tissue responses to CSCs in an environment and conditions similar to reality, constituting a good intermediate point between in vitro and in vivo experimentation [17]. Since its description, numerous publications have employed this model to test histologically different calcium silicate materials, both commercial and experimental. However, available studies vary considerably in culture duration, experimental conditions, and histological assessment methods, limiting comparability across reports.

Accordingly, the aim of the present systematic review is to analyze and synthesize the evidence on direct pulp capping with CSCs using the natural tooth culture model, focusing on experimental conditions as well as histological and immunohistochemical outcomes.

## 2. Materials and Methods

### 2.1. Guidelines and Protocol

This review was conducted in accordance with the PRISMA 2020 preferred reporting guidelines for systematic reviews (Supplementary Materials: PRISMA_2020_checklist-JFB2026) [24]. The review protocol was prospectively registered in the Open Science Framework (OSF) (Registration DOI 10.17605/OSF.IO/8KUX3).

### 2.2. Review Question

This review addressed the histological responses induced in the dental pulp by CSCs when the ex vivo human natural tooth culture model is used as the experimental system. The research question was structured according to the PICO framework, considering as P (population) ex vivo human natural tooth culture models with direct pulp exposure; I (intervention) the application of CSCs, either commercial or experimental, used as direct pulp capping agents; C (comparison) cultured teeth without material application, when available; and O (outcomes) the histological findings described in the pulp tissue, with or without complementary analyses such as immunohistochemistry (IHC), scanning electron microscopy (SEM), energy-dispersive X-ray spectroscopy (EDX), or micro-computed tomography (µCT). Therefore, the wording of the review question and focus was as follows: “In ex vivo human natural tooth culture models, does direct pulp capping with CSCs, compared with other pulp-capping materials or no material, result in different histological tissue responses?”

### 2.3. Search Strategy

A systematic search of electronic databases (PubMed, Embase, Scopus, and Google Scholar) was conducted from database inception to October 2025. No language restrictions were applied. Detailed search strings and any database-specific adaptations are provided in Supplementary Table S1. In addition, grey literature was screened (reference lists of included studies and related reviews; contact with field experts) to minimize publication bias.

### 2.4. Inclusion and Exclusion Criteria

Inclusion criteria: Ex vivo studies using the whole human natural tooth culture model with direct pulp exposure; studies testing CSCs as direct pulp capping agents (commercial or experimental); histological assessment of pulp tissue (H&E and trichrome), with or without complementary analyses (IHC, SEM, EDX, µCT); and original studies published in peer-reviewed journals.

Exclusion criteria: In vitro cell-only studies or animal models (no entire tooth model), studies focused solely on physicochemical/mechanical properties without histological evaluation, reviews/letters/abstracts or incomplete reports, duplicate publications (the most complete version was retained), and studies with no group including a calcium silicate-based material.

### 2.5. Study Selection

Four reviewers (A.C.-F., H.G.d.S.C., D.B.S., J.M.M.d.S.) participated in the selection process. Two reviewers independently screened titles/abstracts and full texts against the eligibility criteria (A.C.-F. and H.G.d.S.C.). A third author resolved any disagreements (J.M.M.d.S.). Rayyan app (available at https://new.rayyan.ai/reviews, accessed on 11 January 2026, Rayyan, Qatar Computing Research Institute, Qatar Foundation) was used to manage the screening workflow and to perform duplicate removal; Mendeley Desktop software (version 1.19.8, Elsevier Inc., New York, NY, USA) was used as the reference manager for organizing the final set of included studies. After full-text assessment, the final selection was established.

### 2.6. Data Extraction

Two independent reviewers (A.C.-F., A.D.-C.) extracted the following variables using a pre-piloted form: first author, tooth type, sample size, culture duration, culture medium, material tested (commercial/experimental), histological findings, immunohistochemical findings, and mineralization outcomes. Discrepancies were resolved through discussion and, when needed, by a third reviewer (J.M.M.d.S.); original articles were consulted to adjudicate uncertainties. When required information could not be retrieved from the publication, corresponding authors were contacted; if data remained unavailable, the study was excluded from quantitative synthesis for that outcome. The accuracy of the extracted data was verified.

### 2.7. Quality Assessment and Risk of Bias of Individual Studies

Risk of bias was assessed using the QUIN Tool (for in vitro dental research) [25] which is applicable to ex vivo whole-tooth culture models. The tool comprises 12 items: (1) clearly stated aims/objectives; (2) sample size calculation/justification; (3) sampling technique with inclusion/exclusion criteria; (4) details of the comparison/control group; (5) methodological detail and standardization; (6) operator details (training/calibration); (7) randomization; (8) method of measuring outcomes (procedure/justification); (9) outcome assessor details (training/calibration, inter/intra-rater reliability where applicable); (10) blinding (operators/evaluators/statistician, as applicable); (11) statistical analysis (software/methods); and (12) presentation of results. Each item was scored as 2 = adequately specified, 1 = inadequately specified, or 0 = not specified; not applicable (NA) items were excluded from the denominator. The QUIN % was calculated as (sum of points × 100)/[2 × number of applicable items] and used to classify studies as low (>70%), moderate (50–70%), or high (<50%) risk of bias. Two reviewers (A.C.-F. and D.B.S.) performed assessments independently, resolving disagreements by consensus or third-party adjudication (J.M.M.d.S.). Corresponding summary tables are provided in Supplementary Table S2 (criteria) and Supplementary Table S3 (study-level ratings).

## 3. Results

### 3.1. Study Selection

**Figure 1:**
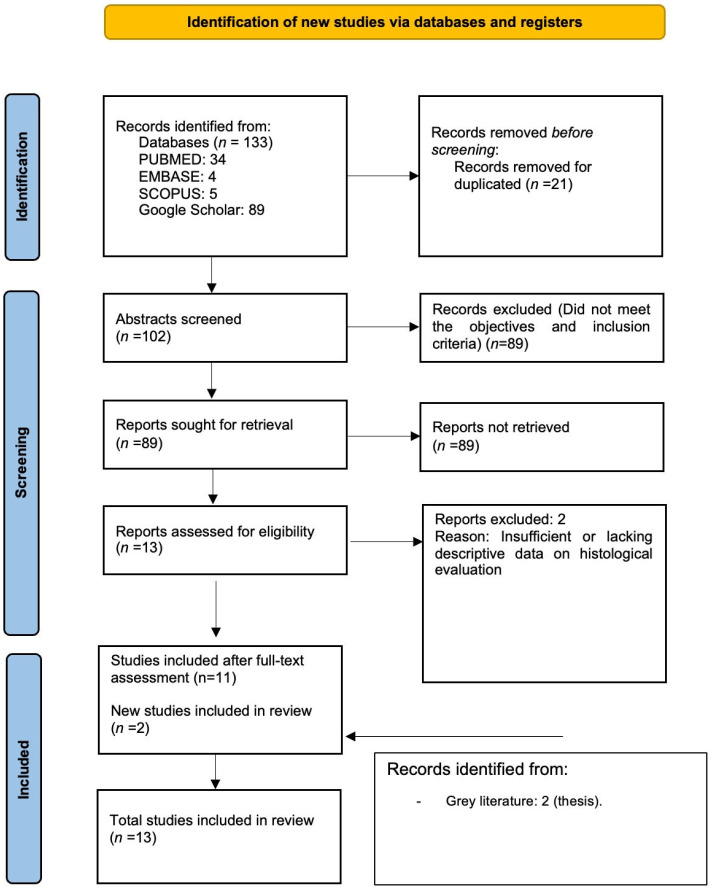
Flowchart of the search strategy and steps of this systematic review.

Four electronic databases—PubMed (34 records), Embase (4), Scopus (5), and Google Scholar (89)—were searched using a multistep strategy. Across all sources, 133 records were retrieved. After removing duplicates, 102 records remained for screening. Subsequently, 89 records were excluded for not meeting the prespecified inclusion criteria, leaving 13 records for full-text assessment. Of these, 2 were excluded due to insufficient or missing descriptive information in the histological assessment [26,27]. Two additional theses were identified through a hand search of the grey literature, resulting in 13 records included in the review [23,28,29,30,31,32,33,34,35,36,37,38,39]. The flowchart of the search process is displayed in Figure 1.

### 3.2. Risk of Bias in the Included Studies

All included studies were assessed for risk of bias using the QUIN tool (Supplementary Tables S2 and S3). Using the prespecified thresholds, 12 of 13 studies (92.31%) were rated as having moderate risk of bias and 1 of 13 (7.69%) as low risk; none were rated as high risk. Overall reporting was adequate across most domains. All studies clearly stated their aims/objectives, described the sampling technique and methodological approach, and presented their results with sufficient clarity. Outcome measurement was adequately described in all studies except one [36]. Comparison groups were reported in most studies; however, two raised concerns regarding interpretability [34,35], and one was deemed not applicable [28].

In contrast, reporting of operator-related information was limited: six studies provided partial information [29,30,31,32,33,39], whereas seven did not report this domain [23,28,34,35,36,37,38]. Randomization and blinding were seldom described adequately: only one study reported each domain in sufficient detail [38], with the others providing insufficient or no information. Reporting outcome assessor characteristics was also incomplete: four studies provided partial information [30,31,35,38], and none reported this domain comprehensively. Statistical analysis was usually described, with one study omitting this item [34].

### 3.3. Data Extraction

Across the included studies, histological outcomes were reported for several commercial CSCs (Angelus MTA, Biodentine, iRoot BP Plus Putty, Neo MTA Plus, ProRoot MTA, TheraCal LC, TotalFill, White ProRoot MTA), with Biodentine being the most frequently characterized. In addition, multiple experimental calcium silicate-containing formulations were also tested (Table 1) [30,31,33,35,37]. Most studies used immature third molars extracted for orthodontic reasons from patients in the age range of 15–19; exceptions were Al-Saludi et al. [32], who used mature premolars with apical sectioning, and Khazane et al. (2022) [36], who included immature premolars in addition to third molars. The Experimental Protocol for Establishing and Maintaining Ex Vivo Whole-Tooth Cultures is presented as Supplementary Table S4.

Routine histology employed hematoxylin–eosin staining, whereas Kuo et al. (2021) [37] additionally used Masson’s trichrome. Immunohistochemistry was performed by Téclès et al. (2008) [23]; Laurent et al. (2012) [28]; and Pedano et al. (2021) [31] (markers included dentine sialophosphoprotein, nestin, type I collagen, and osteonectin).

Regarding follow-up, most studies set a maximum culture/observation period of 28 days [23,28,29,30,31,34,35]. Khazane et al. extended follow-up to 45 days [36] and Al-Saudi et al. to 90 days [32].

**Figure 2:**
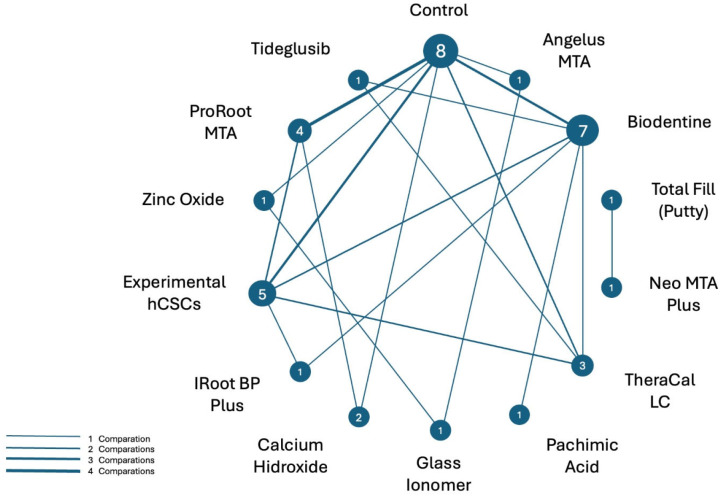
Network diagram of all direct biomaterial comparisons assessed. Nodes represent the biomaterials, and edges (labelled by the number of studies) indicate how frequently each comparison was evaluated.

Figure 2 presents a network diagram of all direct biomaterial comparisons. Nodes represent the biomaterials included in the trials, and edges (labelled by the number of studies) indicate how frequently each comparison was evaluated. Biodentine is the most frequently studied biomaterial, followed by ProRoot MTA and various experimental hCSCs.

### 3.4. Histological and Immunohistochemical Observations

Across hydraulic calcium silicate-based cements (hCSCs), Biodentine showed the most prominent and reproducible early pro-mineralization pattern: several studies described a distinct interface zone (reported as a 50–100 μm amorphous eosinophilic/collagenous band) that delineated the exposure site and was consistently associated with multiple mineralized foci beneath it. Biodentine-associated mineralization could be detected very early (e.g., small amorphous mineralized foci from day 2, progressing to more numerous osteodentin-like foci with cavities/entrapped cells by days 14–28), and when assessed immunohistochemically, these foci frequently showed nestin positivity with DSPP/collagen I/osteonectin expression consistent with odontoblast-like differentiation within/around the mineralized structures. In longer culture conditions, Biodentine was also associated with more advanced repair with minimal inflammatory infiltration and a more organized odontoblast-like layer [30,34,35,36,37,39]. ProRoot MTA and experimental hCSCs similarly maintained a generally well-organized pulp and induced periexposure mineralized foci containing entrapped cells, with classic observations of a thin acellular necrotic interface zone and early osteodentin-like foci after MTA, and entrapped cells within foci in both MTA, TCS 50 and putty-type bioceramics (e.g., TotalFill, Neo MTA Plus) [23,32,33,37].

In contrast, the resin-modified calcium silicate-based cement (Rm-CSC) TheraCal LC showed a more variable and often delayed hard-tissue response: some models reported no pulpal reaction/no mineralized foci at earlier time points (14 days), whereas later evaluations reported small or less frequent mineralized foci [31,33,34]. For experimental Rm-CSCs, histology was strongly formulation-dependent and heterogeneous [31]. Other materials evaluated—pachymic acid, tideglusib, zinc oxide–eugenol, glass ionomer cement and the classical calcium hydroxide—generally exhibited less favourable histological performance than the reference hCSCs [34,36,38].

## 4. Discussion

This systematic review investigated ex vivo human tooth culture models, analyzing the histological, immunohistochemical, and biomineralization effects of CSCs used in direct pulp capping procedures. The findings, consistent with histological and immunohistochemical evidence derived from the intact human tooth culture model, confirm that hCSCs are biocompatible materials with notable bioactive potential.

Across all studies that characterized the histological response to commercially available, resin-free CSCs, researchers consistently reported early mineralization nodules and, in some cases, formation of a dentinal bridge over the follow-up period [23,28,29,30,32,33,34,35,36,37,38,39].

These observations were complemented by in vitro cell culture studies. Reis et al. (2021) demonstrated the viability of dental pulp stem cells (DPSCs) cultured in media conditioned with Biodentine extracts (1:1), showing no significant differences from controls at 24, 48 and 72 h [39]. Comparable results were reported by Sequeira et al. (2018), who examined the viability of stem cells from the apical papilla (SCAP) cultured in media conditioned with ProRoot MTA (1:1) [40]. In addition, Kuo et al. (2021) reproduced this model in pigs using an equivalent experimental protocol and obtained congruent results with the entire tooth model, showing mineralization foci in both experimental models [37].

The mineralization foci observed are associated with the deposition of an irregular, atubular mineralized matrix referred to as osteodentine, which serves as a key precursor in reparative dentinogenesis [41]. In this regard, Téclès et al. (2008), Laurent et al. (2012), and Pedano et al. (2021) complemented histological characterization with immunohistochemistry, reporting expression of type I collagen (COL-I), nestin (NES), dentine sialophosphoprotein (DSPP) and osteonectin (SPARC) [23,28,31]. The presence of proteins such as NES supports the notion that this represents an incipient form of reparative dentine, as NES is a marker of secretory human odontoblasts [42]. Likewise, DSPP expression is essential for dentine formation by regulating mineralization [43].

From a biological perspective, the localization of mineralization nodules near pulpal microvasculature may reflect the involvement of perivascular mesenchymal progenitor populations. Histological observations in the included studies frequently showed mineralized nodules located in proximity to pulpal microvascular structures. Perivascular niches are recognized sources of multipotent mesenchymal stem/stromal cells, and their presence has been demonstrated in vascular walls and microvessels, where they exhibit multilineage differentiation potential, including osteogenic commitment [44]. Moreover, mesenchymal stem cell populations have been shown to remain viable in inflamed periapical tissues, suggesting that these progenitors can actively participate in bone formation and repair processes under inflammatory conditions [45]. In parallel, several studies have reported that bioactive materials, including calcium silicate-based biomaterials, can promote mesenchymal stem cell differentiation toward mineralizing phenotypes, further supporting their role in hard tissue regeneration [46,47]. Taken together, these findings suggest that perivascular stem cell populations present within pulpal microvasculature may contribute to the pool of progenitor cells that migrate toward injury sites, differentiate into odontoblast-like cells, and participate in the formation of mineralized nodules resembling osteodentin.

Stimulation of reparative dentine formation by hCSCs correlates with their ability to induce the release of several cytokines and growth factors, notably BMPs, TGF-β1, VEGF, FGF-2 and PDGF [48]. Laurent et al. showed that Biodentine significantly increased TGF-β1 secretion by pulp cells [28]. This growth factor modulates progenitor-cell migration and differentiation, thereby contributing to reparative dentine formation. The cytokine stimulatory effect has been attributed to the release of calcium hydroxide Ca(OH)_2_ during material setting, which dissociates into OH^−^ and Ca^2+^ ions. Ionic release has thus been described as a key driver of DPSC migration, proliferation and differentiation [49]. Consistently, extracellular Ca^2+^ has been shown to promote odontoblastic differentiation of DPSCs via the BMP2–Smad1/5/8 and Erk1/2 pathways [50].

In the present review, mineralization induced by ProRoot MTA was more homogeneous than that produced by the classical pulp-capping material Ca(OH)_2_ [23]. These data are comparable to the in vivo findings of Aeinehchi et al. [51]. Moreover, Ricucci et al. demonstrated that major causes of failure with Ca(OH)_2_-based pulp capping materials include tunnel defects within the dentinal bridge and progressive dissolution of the material over time [52]. In line with this, Peng et al. (2011) concluded that hCSCs promote greater DPSC proliferation and odontogenic differentiation than Ca(OH)_2_ [53]. A recent meta-analysis likewise showed the clinical superiority of hCSCs over traditional calcium hydroxide pastes, reporting higher success rates at all follow-up points (6, 12 and 36 months), with overall success ranging from 80% to 100% even after three years [54].

Biodentine was the most extensively characterized biomaterial within this model. Introduced in 2011 as an alternative to ProRoot MTA, it is an hCSC primarily composed of tricalcium silicate (C_3_S) and dicalcium silicate (C_2_S), with zirconium oxide (ZrO_2_) as radiopacifier [55]. In all studies evaluating Biodentine, mineralization nodules were observed; notably, Laurent et al. (2012) reported Biodentine particles embedded within these nodules, which further supports the material’s mineralization potential [28]. Clinical studies have corroborated these experimental findings, supporting the mineralization potential and biocompatibility of both Biodentine and ProRoot MTA [11].

Premixed hydraulic calcium silicate-based cements (hCSCs) represent a more recent alternative to conventional powder–liquid formulations, characterized by their ready-to-use presentation and simplified clinical handling. The development of fast-setting putty-type formulations has further expanded their applicability, as their cohesive consistency and improved resistance to wash-out make them suitable for a wide range of procedures, including pulpotomies and surgical endodontic interventions. Initially introduced in 2007 under different commercial names sharing similar compositions, these materials have evolved toward faster-setting premixed putty formulations with enhanced stability in moist environments [14]. Some of these materials, such as EndoSequence and TotalFill putties, achieve their composition through the incorporation of monobasic calcium phosphate into calcium silicate matrices, together with polymeric vehicles that modulate handling and setting behaviour [56].

Several studies have biologically characterized these premixed putty materials, reporting responses comparable to those of classical formulations. For instance, Lozano-Guillén et al. demonstrated similar cell viability between NeoPutty and MTA Angelus at ½ and ¼ dilutions using MTT assays, without statistically significant differences, and comparable results in cell migration tests [57]. Likewise, Cruz Hondares et al. observed no significant differences in biological behaviour between NeoPutty and EndoSequence when compared with conventional hCSCs such as ProRoot MTA and MTA Angelus [58]. These findings are consistent with those obtained using the intact tooth culture model, where Al-Saudi et al. (2019) reported complete mineralization at 90 days with TotalFill putty [32]. Clinical evidence further supports the performance of premixed hCSCs: a recent systematic review and meta-analysis demonstrated their effectiveness as pulpotomy materials in both primary and permanent teeth, with success rates comparable to MTA and reduced risk of coronal discoloration. Similar outcomes were reported by Ghaly et al. (2025), who found Well-Root PT to be effective in apexification procedures when compared with MTA [59].

From a physicochemical standpoint, Minju Song et al. showed that putty-type cements exhibit chemomechanical properties, biocompatibility, and mineralization potential comparable to ProRoot MTA, supporting the concept that their modified presentation does not compromise their bioactive performance [60].

Resin-modified CSCs are now available and offer improved clinical handling, enhanced sealing and dentine adhesion, compatibility with restorative materials, and better mechanical properties [31]. One such material is TheraCal LC. Sukajintanakarn et al. and X. Li et al. reported small, amorphous and fewer mineralization foci in histological sections [33,34]. Similarly, Pedano et al. observed a complete absence of early mineral formations [31]. Overall, these findings indicate that resin-modified CSCs demonstrate inferior histological performance, showing reduced or delayed mineralization when compared with reference materials such as Biodentine and ProRoot MTA. This may relate to the high resin content, which could polymerize incompletely and leach toxic monomers into the pulp, negatively affecting cell viability, proliferation and migration findings that are concordant with in vitro [61] and clinical studies [15]. In the work by Pedano et al., an experimental resin-containing hCSC (“Exp_HEAA”) provided favourable outcomes in terms of early mineralization and nodule formation [31]. Its organic phase incorporated a hydrophilic monomer (HEAA) that promoted hydration of calcium silicates, alongside other monomers that facilitated polymerization, thereby reducing cytotoxicity and enabling early mineralization with NES and COL-I expression. Two additional experimental hCSCs also showed promising histological outcomes: “TCS 50”, which used a pure C_3_S-based CSC with 50% ZrO_2_ added [33], and “RDSC”, a sol–gel synthesized CSC employing Bi_2_O_3_ as a radiopacifier [37].

Although several of the included studies reported numerical or semi-quantitative findings related to mineralized tissue formation, the marked heterogeneity in experimental design, outcome definitions, scoring systems, and evaluation time points precluded meaningful quantitative synthesis or direct comparisons between studies. Reported measures ranged from semi-quantitative histological scores to morphometric analyses and counts of mineralized foci, often obtained under different culture durations and methodological conditions. For example, Somudorn et al. reported higher histological scores for mineralized tissue formation in samples treated with calcium silicate-based materials (Biodentine) compared with other groups, whereas Kumar et al. used a semi-quantitative grading system for reparative responses, showing increased mineralized barrier formation in the Biodentine-treated group [35,36]. Additionally, Pedano et al. described the percentage of teeth exhibiting mineralized foci, while Duarte et al. presented semi-quantitative comparisons of hard tissue deposition associated with the evaluated materials and the degree of histological pulp involvement [31,38]. Despite these numerical observations, the lack of standardized outcome measures and differing evaluation criteria prevent reliable data aggregation or frequency-based comparisons. Nevertheless, these individual quantitative findings consistently support the trend toward early mineralized tissue deposition and odontoblast-like cell differentiation within this ex vivo model when calcium silicate-based materials are used.

The experimental model employed throughout was the intact human tooth culture. This represents an intermediate approach between in vitro cell cultures and in vivo animal models. Most in vitro cultures are two-dimensional, and cells lack their native structural arrangement and physiological interactions. Although animal experimentation remains the current benchmark in material development, interspecies differences may yield divergent tissue responses [17]. In this context, the intact human tooth culture is a valuable tool because cells remain in their physiological state under near natural conditions. It is therefore a cost-effective, rapid and reproducible methodology to investigate cellular mechanisms following pulp exposure. Histological assessment is also the most reliable method to evaluate pulp status, as it enables grading of inflammation and detection of necrosis [32].

However, the model has limitations. Although the ex vivo natural tooth culture model provides relevant information on the interaction between the dentin–pulp complex and calcium silicate-based biomaterials, as it preserves tissue cytoarchitecture and cell–extracellular matrix interactions [17], its inherent limitations must be acknowledged. First, this experimental system primarily enables the assessment of early pulp tissue responses, providing a histological snapshot of a specific culture time point, but without the possibility of dynamically monitoring the temporal evolution of the inflammatory–reparative process [33].

Moreover, because the model is isolated from systemic circulation, the lack of vascular perfusion and functional blood supply may affect tissue metabolism (e.g., hypoxia-related changes), thereby influencing the magnitude and complexity of inflammatory and reparative responses. Under these conditions, inflammatory reactions may be incomplete or limited, which should be considered when extrapolating findings to clinical scenarios. This limitation may also contribute to morphological differences in the resulting mineralized deposits, such as the absence of the typical tubular morphology described under more physiological conditions [28]. In addition, most ex vivo systems are established under “ideal” conditions of healthy pulp of immature teeth, without incorporating clinically relevant variables such as pre-existing pulp inflammation or bacterial invasion, which could substantially alter tissue responses to biomaterials; therefore, the outcomes observed in this model should be interpreted within this specific experimental context. Finally, this model is particularly suitable for investigating vital pulp therapy procedures, but it is not fully appropriate for regenerative endodontic procedures, as these require necrotic pulp conditions and interventions such as partial tissue removal, which may not meet the methodological criteria proposed for a true ex vivo model [17].

Considering these limitations, ex vivo models based on extracted teeth are especially useful for investigating dentin–pulp complex responses to biomaterials in vital pulp therapy procedures, as they enable tissue-level assessment within a preserved anatomical context. In contrast, in vitro models, including both 2D and 3D systems, are particularly valuable during early phases of biomaterials research due to their speed, low cost, and high experimental control, allowing the investigation of specific mechanisms (cytocompatibility, ion release, cell differentiation, and mineralization-related markers). Nonetheless, because they have limited capacity to replicate tissue complexity and clinically relevant pathophysiological conditions, they should be complemented by models that better preserve pulp architecture and microenvironmental cues. Finally, in vivo models represent the most physiologically relevant preclinical approach, as they incorporate vascular perfusion, immune responses, and reparative processes under complete biological conditions. However, they also involve substantial challenges, including higher cost, technical complexity, ethical considerations, and interspecies variability; therefore, they are generally reserved for advanced preclinical stages prior to translation into human studies [17].

Culture duration should also be considered: Pedano et al. showed that, even four weeks after extraction, third-molar pulps retained similar behaviours and were capable of expressing markers comparable to stem cells from freshly extracted teeth, supporting the validity of 28-day culture periods to study early mineralization phases [29]. However, studies by Kuo et al. and Al-Saudi et al. extended culture to 45 and 90 days, respectively [32,37], which may allow better characterization of CSCs’ influence on reparative dentinogenesis. Moreover, future studies could benefit from the incorporation of advanced analytical approaches beyond conventional histological and immunohistochemical assessment. Techniques such as iterative multiplex immunofluorescence and miRNA profiling may provide more comprehensive insight into the molecular and cellular responses of pulp tissue to emerging biomaterials. In addition, recent advances in transcriptomic and spatially resolved molecular analyses have been successfully applied to characterize the complex cellular landscape and spatial organization of dental tissues, particularly in developmental contexts, highlighting their potential for broader application in dental research. These unbiased and spatially contextualized approaches may offer deeper understanding of tissue heterogeneity, cell–cell interactions, and repair-associated signalling pathways [62].

In this context, the intact tooth ex vivo culture model may represent a particularly suitable platform for integrating such molecular strategies, as it preserves tissue architecture while allowing controlled experimental manipulation. Furthermore, tissue-clearing techniques combined with three-dimensional imaging have emerged as powerful tools in dental research, enabling volumetric visualization of soft and hard tissues, neurovascular networks, and mineralized structures without the limitations of conventional two-dimensional sectioning. These approaches may facilitate spatially resolved evaluation of pulp responses and hard tissue formation throughout the entire dentin–pulp complex, thereby expanding the analytical potential of ex vivo tooth culture systems [63].

Regarding the limitations of this review, most included articles presented a moderate risk of bias according to the applied assessment tool (QUIN Tool). This level of risk does not invalidate the reported findings but suggests that certain methodological aspects—such as sample size calculation, operator details, or outcome assessor details—may not have been consistently controlled across studies. Consequently, the results should be interpreted as indicative of general biological trends rather than as precise quantitative estimates of material performance.

The heterogeneity of materials and methodological variability hinders direct comparison across studies. Moreover, only non-carious molars and premolars were included, which limits generalizability and leaves open the question of whether the lack of pre-existing pulpal inflammation influenced the observed outcomes.

From a clinical perspective, the histological findings observed in this model help anticipate the early pulp tissue responses that may occur following vital pulp therapy procedures involving direct pulp exposure. The intact tooth ex vivo culture system reproduces a controlled pulpal injury and allows evaluation of the subsequent reparative processes triggered by biomaterial application, including mineralized tissue deposition and odontoblast-like cell differentiation. Within these experimental conditions, calcium silicate-based materials without resin components consistently demonstrated more organized pulp tissue architecture and more pronounced early mineralized tissue formation compared with resin-modified formulations. While clinical outcomes depend on multiple additional factors, these tissue-level observations support the use of conventional calcium silicate cements as reliable biomaterials for promoting favourable reparative responses in vital pulp therapy.

## 5. Conclusions

Hydraulic CSCs can stimulate reparative dentinogenesis, thereby helping to preserve the integrity of the dentine–pulp complex through localized remineralization at the injury site. Histological analysis of pulp sections across the included studies supports this conclusion. The intact human tooth model emerges as a reliable, economical and reproducible approach to examine the early stages of the pulp tissue reparative process. However, further studies using longer culture/observation periods and larger sample sizes are needed to strengthen the evidence base and to better establish this model as a screening platform for pulp-capping materials.

## Figures and Tables

**Table 1:** Summary of included studies: design/features and histological, immunohistochemical, and mineralization findings.

First Author, Year Published	Tooth Type; Sample Size	Culture Time (Days)	Culture Medium	Material Tested	Histological Results	Immunohistochemical Results	Mineralization
Téclès et al., 2008 [23]	Immature third molars (1/3 or 2/3 root); 35	1, 14, 28	MEM + 10% FBS + 2 mM β-GP	Control	Normal pulp with slight fibrous condensation at the exposure site.	_	Absent
				Calcium hydroxide XR	Day 1: thick acellular necrotic zone was observed directly beneath the biomaterial. Days 14–28: appearance of foci containing sequestered cells and with heterogeneous size.	Dentin sialoprotein immuno-labelling was similar to collagen I expression. It was localized in the pulp matrix, mineralized foci matrix and in the sequestered cells	Present from day 1, localized, heterogeneous, and with higher distance to the exposure site.
				ProRoot MTA	Day 1: thin acellular necrotic zone beneath the material, with dense connective tissue and early mineralized foci (osteodentin). Days 14–28: increased mineralized foci beneath the material.	Strong expression of type I collagen in the matrix of mineralized foci; DSPP and nestin expressed in entrapped cells and in both the pulp and mineralized foci matrices, consistent with odontoblastic differentiation.	Present from day 1, localized, homogeneous, and closer to the exposure site.
Duarte et al., 2010 (Thesis) [38]	Immature third molars (1/3 or 2/3 root); 45	1, 14, 28	DMEM + 10% FBS + 1% (P/S + Aβ)	Control	Day 1: moderate chronic inflammation, moderate hyperemia, necrosis absent, and absence of an odontoblastic layer. Day 14: similar pattern with partial formation of mineralized structure. Day 28: moderate mixed inflammation, mild hyperemia, no necrosis or odontoblastic layer.	_	Partial mineralization at 14 days and focal at 28 days; mean thickness (μm): 18.8 (1 d), 25.7 (14 d), 38.2 (28 d).
				Calcium hydroxide PA	Day 1: moderate chronic inflammation, severe hyperemia, absence of necrosis and presence of edema. Day 14: moderate inflammation, severe hyperemia, no necrosis. Day 28: moderate chronic inflammation hiperemia.		Mineralization initiated at day 1, complete at 14 and 28 days; Mean thickness (μm): 21.3 (1 d), 55.6 (14 d), 96.5 (28 d).
				Zinc oxide eugenol cement	Day 1: moderate chronic inflammation, absence of necrosis and presence of edema. Day 14: moderate inflammation, no necrosis. Day 28: moderate chronic inflammation.		Mineralization initiated at day 1, partial at 14 and 28 days; Mean thickness (μm): 31.9 (1 d), 27.8 (14 d), 21.9 (28 d).
				Glass ionomer cement	Day 1: mild chronic inflammation and presence of edema. Day 14: moderate inflammation, severe hyperemia, no necrosis. Day 28: severe chronic inflammation and moderate hiperemia.		Mineralization initiated at day 1, complete at 14 and 28 days; Mean thickness (μm): 23.6 (1 d), 45.3 (14 d), 37.8 (28 d).
				Angelus MTA	Day 1: moderate chronic inflammation, necrosis present and edema. Day 14: moderate inflammation and no necrosis. Day 28: moderate chronic inflammation, no necrosis or odontoblastic layer.	_	Mineralization initiated at day 1, partial at 14 days, and complete at 28 days; Mean thickness (μm): 32.4 (1 d), 50.0 (14 d), 26.3 (28 d).
Laurent et al., 2012 [28]	Immature third molars (1/3 or 2/3 root); 15	2, 14, 28	DMEM + 10% FBS + P/S (200 U/mL, 200 µg/mL) + Aβ (0.5 µg/mL)	Biodentine	Day 2: small amorphous mineralized foci in the pulp connective tissue adjacent to the material (2/5 samples). Days 14–28: increased number of mineralized foci in all samples, containing cavities with entrapped cells and an osteodentin-like appearance.	DSPP expressed in the mineralized matrix and entrapped cells; nestin positive in mineralized foci; type I collagen and osteonectin also expressed.	Early mineralization (2 days), progressive, forming non-tubular osteodentin foci.
Al-Saudi et al., 2019 [32]	Premolars with apical sectioning (~3 mm) to create an artificial opening; 40	21, 90	DMEN + P/S (300 UI/mL, 300 µg/mL) + Aβ (0.75 µg/mL)	TotalFill (Putty)	Day 21: partial dentin bridge formation in most samples (9/10), with no signs of pulpal inflammation (8/10); congested vessels, occasional V-shaped reparative dentin and pulp stones. Day 90: complete dentin bridge formation (10/10), homogeneous, dense, and tubular in appearance; absence of pulpal inflammation (10/10).	_	Partial mineralization at 21 days (0.1–0.25 mm) and complete at 90 days (>0.25 mm in 100% of samples).
				Neo MTA Plus	Day 21: partial dentin bridge formation (8/10), no signs of pulpal inflammation (7/10); irregular pattern of reparative dentin, superficial necrosis, and congested vessels. Day 90: complete dentin bridge formation (10/10), no pulpal inflammation (10/10); bridge separated by a demarcation line, thin and atubular.	_	Partial mineralization at 21 days (0.1–0.25 mm) and complete at 90 days in all samples; >0.25 mm in 60% and 0.1–0.25 mm in 40%.
Pedano et al., 2019 [29]	Immature third molars (1/3 or 2/3 root); 6	7, 14, 28	DMEM + 10% FBS + 1% P/S + 1% Aβ	Control	Pulp tissue with normal architecture and cell distribution, without eosinophilic band.	_	Absent
				ProRoot MTA	Continuous collagen band at the pulp–material interface, with periexposure mineralized foci containing entrapped cells.	_	Mineralized foci at the exposure site after 4 weeks.
Pedano et al., 2020 [30]	Immature third molars (1/3 or 2/3 root); 9	28	DMEM + 10% FBS + 1% P/S + 1% Aβ	Control	Well-organized pulp tissue with normal histological features, showing no inflammatory reaction or mineralized foci.	_	Absent
				Biodentine	Well-structured pulp tissue with a 50–100 μm fibrinous hyaline band delineating the exposure area, beneath which multiple mineralized foci were observed.	_	Presence of multiple mineralized foci after 4 weeks.
				PPL-hCSC (Experimental)	Amorphous eosinophilic layer at the pulp–material interface, with numerous adjacent mineralized foci.	_	Mineralized foci extending 20–50 μm into the pulp tissue.
Sukajintanakarn et al., 2020 [34]	Immature third molars (1/3 or 2/3 root); 36	14, 28	DMEM + 10% FBS + P/S (200 U/mL, 200 µg/mL) + Aβ (0.5 µg/mL)	Tideglusib	Well-preserved pulp tissue with no eosinophilic areas, inflammation, vascular changes, or mineralized foci at 14 and 28 days.	_	Absent
				Biodentine	Amorphous eosinophilic zone at the interface with the biomaterial; pulp tissue without inflammation and with multiple mineralized foci.	_	Evident mineralized foci at 14 and 28 days.
				Theracal LC	14 days: no pulpal reaction observed. 28 days: small mineralized foci adjacent to the material, with no increase in vascularity or inflammation.	_	Small mineralized foci at 14 and 28 days.
Xin Li et al., 2020 [33]	Immature third molars (1/3 or 2/3 root); 24	14, 28	DMEM + 10% FBS + P/S (200 U/mL, 200 µg/mL)	Control	Normal cell morphology with a superficial necrotic layer and underlying connective tissue; no evidence of mineralization or healing.	_	Absent
				ProRoot MTA	14 days: formation of mineralized foci. 28 days: mineralized foci similar in number and morphology to those at week 2, with entrapped cells inside.	_	Mineralized foci with osteodentin-like morphology at 2 and 4 weeks.
				Theracal LC	14 days: no mineralized foci observed. 28 days: mineralized foci present in 2/3 of samples, showing morphology and size comparable to other materials.	_	Late and less frequent formation of mineralized foci.
				TCS 50 (Experimental)	14 days: formation of mineralized foci of variable size and irregular shape, containing entrapped cells; well-organized pulp tissue. 28 days: mineralized foci similar in morphology and number to those at week 2, with entrapped cells within the matrix.	_	Irregular mineralized foci with entrapped cells at 2 and 4 weeks.
Reis et al., 2021 (Thesis) [39]	Immature third molars (1/3 or 2/3 root); 11	7	KO-DMEM + 20% FBS + P/S (100 U/mL, 100 μg/mL) + GlutaMAX + β-mercaptoethanol	Biodentine	Eosinophilic layer consistent with a collagen band separating the material from the pulp tissue; mild inflammatory reaction, presence of mineralized foci, increased neovascularization near the exposure site, and slight edema.	_	Multiple mineralized foci in the pulp lumen, consistent with osteodentin that will develop into reparative dentin.
Pedano et al., 2021 [31]	Immature third molars (1/3 or 2/3 root); 10	14	DMEM + 10% FBS + 1% P/S + 1% Aβ	Control	No tissue reaction, with absence of mineralized foci and cell migration.	Nestin: negative; type I collagen: negative.	Absent
				Theracal LC	No pulpal tissue reaction, absence of mineralized foci, and presence of fibrous tissue.	No fibrinous layer or mineralized foci observed; no pulpal tissue reaction.	Absent
				HEAA (Experimental)	Exposure area rich in collagen fibrils and capillaries, showing an amorphous eosinophilic layer beneath which numerous mineralized foci were observed.	Mineralized foci strongly positive for nestin and less intensely stained for type I collagen, indicative of dentin matrix formation.	Mineralized foci (20–50 μm) at the exposure area, confirmed by µCT and histology.
				GDM (Experimental)	Absence of eosinophilic layer and no formation of mineralized foci.	Nestin: negative; type I collagen: negative.	Absent
				HEAA/GDM (Experimental)	No mineralized foci or fibrinous tissue reaction observed.	_	Absent
Kuo et al., 2021 [37]	Immature third molars (1/3 or 2/3 root); 45	1, 7, 14	α-MEM + 10% FBS + 1% P/S + 0.75 μg/mL Aβ	Control	Normal pulpal morphology with no evidence of necrosis.	_	Absent
				White Proroot MTA	Mineralized foci near the exposure area, similar in size and number to RDSC, containing entrapped cells. Masson’s trichrome staining revealed a blue-stained area surrounding the mineralized foci, indicating collagen accumulation.	_	Mineralized foci observed near the exposure area.
				Biodentine	Mineralized foci near the exposure area, smaller in size than those in other materials, with entrapped cells present.	_	Small mineralized foci observed near the exposure area.
				iRoot BP Plus Putty	Mineralized foci near the exposure area, containing entrapped cells.	_	Mineralized foci observed near the exposure area.
				RDSC (Experimental hCSC)	Mineralized foci under the exposure area, potentially leading to dentin bridge formation; some contained entrapped cells with an osteodentin-like appearance. Masson’s trichrome staining revealed a blue-stained area surrounding the mineralized foci, indicating collagen accumulation.	_	Formation of mineralized foci.
Khazane et al., 2022 [36]	Immature premolars or third molars (1/3 or 2/3 root); 40	45	DMEN + 5% FBS + P/S (300 UI/mL, 300 µg/mL) + Aβ (0.75 µg/mL)	Pachimic acid	Nonuniform dentin bridge, thinner tan observed in Biodentine group.	_	Dentin bridge thickness predominantly between 0.1–0.25 mm, with a mean thickness of 110 ± 28 µm.
				Biodentine	57% of samples showed an intact dentin bridge; most showed no inflammatory cells or only minimal localized infiltration. A uniform odontoblastic layer with an almost palisade-like arrangement was observed in 57% of samples.	_	50% of samples showed a dentin bridge >0.25 mm thick, with a mean thickness of 134 ± 14 µm.
Somudorn et al., 2023 [35]	Immature third molars (1/3 or 2/3 root); 15	28	DMEM + 10% FBS + P/S + Aβ DMEM + 10% FBS + P/S + Aβ	Control	Typical organization of pulp tissue with slight necrosis at the exposure area and no formation of mineralized tissue or fibrinous reaction.	_	Absent
				Biodentine	Mineralized foci around the exposure area, with amorphous eosinophilic material adjacent to the site; partial necrosis in two cases and mild in one.	_	Mineralized layer adjacent to the pulp capping material.
				ProRoot MTA + GIC (Experimental)	Thin reaction zone between the material and pulp tissue, with mild superficial necrosis in two cases and no mineralized foci.	_	Absent
				Biodentine + GIC (Experimental)	Thin reaction zone between the material and pulp tissue, with mild superficial necrosis in two cases and no mineralized foci.	_	Absent

Abbreviations: Aβ: amphotericin β, α-MEM: alpha minimum essential medium, β-GP: beta-glycerophosphate, DMEN: Dulbecco’s Modified Eagle Medium, DSPP: dentin sialophosphoprotein, GDM: glycerol dimethacrylate + hydraulic calcium silicate-based cement, hCSC: hydraulic calcium silicate-based cement, GIC: glass ionomer cement, HEAA: N-(2-hydroxyethyl) acrylamide + hydraulic bioceramic cement, HEAA/GDM: N-(2-hydroxyethyl) acrylamide + glycerol dimethacrylate + hydraulic bioceramic cement, FBS: fetal bovine serum, MEM: minimum essential medium, MTA: mineral trioxide aggregate, PPL-hCSC: phosphopullulan-functionalized hydraulic calcium silicate-based cement, P/S: penicillin/streptomycin, RDSC: radiopaque dicalcium silicate cement, TCS 50: tricalcium silicate-based cement + 50 wt% ZrO_2._

## Data Availability

The original contributions presented in the study are included in the article; further inquiries can be directed to the corresponding author.

## Supplementary Materials

The following supporting information can be downloaded at https://www.mdpi.com/article/10.3390/jfb17020078/s1, Table S1: Search strategy and eligibility criteria for the systematic search; Table S2: QUIN tool assessment of selected studies; Table S3: Overall evaluation of risk of bias of included studies using the QUIN tool; Table S4: Experimental Protocol for Establishing and Maintaining Ex Vivo Whole-Tooth Cultures; PRISMA_2020_checklist-JFB2026.

## Abbreviations

The following abbreviations are used in this manuscript:

α-MEM	Alpha minimum essential medium
CSCs	Calcium silicate-based cements
DMEM	Dulbecco’s Modified Eagle Medium
DPSCs	Dental pulp stem cells
DSPP	Dentine sialophosphoprotein
FBS	Fetal bovine serum
hCSC	Hydraulic calcium silicate-based cements
Rm-CSC	Resin-modified calcium silicate-based cement
RDSC	Radiopaque dicalcium silicate cement
TCS 50	Tricalcium silicate-based cement + 50 wt% ZrO_2_
VPT	Vital pulp therapy
