# Supplementary material for: Histological Tissue Response to Calcium Silicate-Based Cements Assessed in Human Tooth Culture Models: A Systematic Review
Source: J Funct Biomater. 2026 Feb 6;17(2):78. doi: 10.3390/jfb17020078 (PMC12942347; doi:10.3390/jfb17020078)
Supplement: Supplementary file 1 [file jfb-17-00078-s001.zip › Supplementary Table S1-JMS.pdf]

Supplementary Table S1: Search strategy and eligibility criteria for the systematic search.

**Component**	**Description**
Databases searched	PubMed, Embase, Scopus, Google Scholar
Search period	September 2025
Search terms	((((Calcium silicate cements) OR (Bioactive Endodontic cements)) OR (MTA)) AND (Tooth Culture Model)
Language restrictions	English
Inclusion criteria	- Studies evaluating hydraulic calcium silicate-based materials used as direct pulp capping agents (commercial or experimental formulations).<br>- Experimental models using the human natural tooth culture model with direct pulp exposure.<br>- Histological evaluation of pulp tissue (HE, trichrome, etc.) with or without complementary analyses (IHC, SEM, EDX, μCT).<br>- Studies included regardless of comparator type, provided at least one group tested a calcium silicate-based material (including pulp controls, other silicate materials, or non-silicate comparators).<br>- Original experimental studies published in peer-reviewed journals.
Exclusion criteria	- Animal models or isolated cell cultures (no whole-tooth model).<br>- Studies focused only on physicochemical or mechanical properties without histological evaluation.<br>- Reviews, letters, abstracts, or incomplete reports.<br>- Duplicate publications (the most complete version was selected).<br>- Studies with no group including a calcium silicate-based material.
Screening process	Titles and abstracts screened independently by two reviewers; full texts assessed for eligibility
Data extraction	Standardized extraction of tooth type, culture time, culture medium, material type (commercial/experimental), histological and immunohistochemical findings, and effects on mineralization.
