# Supplementary material for: Histological Tissue Response to Calcium Silicate-Based Cements Assessed in Human Tooth Culture Models: A Systematic Review
Source: J Funct Biomater. 2026 Feb 6;17(2):78. doi: 10.3390/jfb17020078 (PMC12942347; doi:10.3390/jfb17020078)
Supplement: Supplementary file 1 [file jfb-17-00078-s001.zip › Supplementary Table S2-JMS.pdf]

Supplementary Table S2: QUIN tool assessment of selected studies.

First Author; year published	Clearly stated aims/objectives	Detailed sample size calculation	Detailed explanation of sampling technique	Details of comparison group	Detailed explanation of methodology	Operator details	Randomization	Method of measuring outcome	Outcome assessor details	Blinding	Statistical analysis	Presentation of results	TOTAL
Téclès et al., 2007	2	0	2	2	2	0	1	2	0	0	2	2	15
Duarte et al., 2010	2	0	2	2	2	0	2	2	1	2	2	2	19
Laurent et al., 2012	2	0	2	NA	2	0	0	2	0	0	2	2	12
Al Saudi et al ., 2019	2	0	2	2	2	1	1	2	0	0	2	2	16
Pedano et al., 2019	2	0	2	2	2	1	0	2	0	0	2	2	15
Pedano et al., 2020	2	0	2	2	2	1	0	2	1	0	2	2	16
Xin Li et al. 2020	2	0	2	2	2	1	0	2	0	0	2	2	15
Sukajintanakarn et al., 2020	2	0	2	1	2	0	1	2	0	0	0	2	12
Pedano et al. 2021	2	0	2	2	2	1	0	2	1	0	2	2	16
Kuo et al., 2021	2	0	2	2	2	0	1	2	0	0	2	2	15
Reis et al., 2021	2	0	2	2	2	1	0	2	0	0	2	2	15
Khazane et al., 2022	2	0	2	2	2	0	1	1	0	0	2	2	14
Somudorn et al., 2013	2	0	2	1	2	0	0	2	1	0	2	2	14

Abbreviations: NA: Not Applicable.
