# Supplementary material for: Histological Tissue Response to Calcium Silicate-Based Cements Assessed in Human Tooth Culture Models: A Systematic Review
Source: J Funct Biomater. 2026 Feb 6;17(2):78. doi: 10.3390/jfb17020078 (PMC12942347; doi:10.3390/jfb17020078)
Supplement: Supplementary file 1 [file jfb-17-00078-s001.zip › Supplementary Table S3-JMS.pdf]

Supplementary Table S3: Overall evaluation of risk of bias of included studies using the QUIN tool.

First author, year published	Total score	Final score	Risk of bias
Téclès et al., 2007	15/24	62.50	Moderate
Duarte et al., 2010	19/24	79.16	Low
Laurent et al., 2012	12/22	54.55	Moderate
Al Saudi et al ., 2019	16/24	66.67	Moderate
Pedano et al., 2019	15/24	62.50	Moderate
Pedano et al., 2020	16/24	66.67	Moderate
Xin Li et al. 2020	15/24	62.50	Moderate
Sukajintanakarn et al., 2020	12/24	50.00	Moderate
Pedano et al. 2021	16/24	66.67	Moderate
Kuo et al., 2021	15/24	62.50	Moderate
Reis et al., 2021	15/24	62.50	Moderate
Khazane et al., 2022	14/24	58.33	Moderate
Somudorn et al., 2023	14/24	58.33	Moderate
