# Supplementary material for: Histological Tissue Response to Calcium Silicate-Based Cements Assessed in Human Tooth Culture Models: A Systematic Review
Source: J Funct Biomater. 2026 Feb 6;17(2):78. doi: 10.3390/jfb17020078 (PMC12942347; doi:10.3390/jfb17020078)
Supplement: Supplementary file 1 [file jfb-17-00078-s001.zip › Supplementary Table S4-JMS.pdf]

Supplementary Table S4: Experimental Protocol for Establishing and Maintaining Ex Vivo Whole-Tooth Cultures

**1. Sample Selection**	Recommended tooth type: immature human third molars extracted for clinical reasons.<br>Typical age range: 15–20 years.<br>Teeth should present open apices and vital pulp tissue to ensure preservation of pulp architecture and cellular viability.
**2. Transport and Initial Storage**	Immediately after extraction, teeth should be placed in sterile tubes containing 10 mL of DMEM supplemented with 10% fetal bovine serum (FBS), antibiotics (e.g., penicillin–streptomycin), and an antifungal agent (e.g., amphotericin B).<br>Transport time to the laboratory should ideally not exceed 4 hours.
**3. Surface Decontamination and Preparation**	Under sterile conditions, teeth should be briefly rinsed in 70% ethanol (approximately 1 minute), followed by sterile PBS, or alternatively disinfected by immersion in 0.2% chlorhexidine solution and PBS for 10 and 30 seconds, respectively.<br>Residual periodontal ligament tissue should be removed using a sterile scalpel.<br>Teeth should be handled with sterile gauze moistened with culture medium to avoid dehydration.
**4. Cavity Preparation and Pulp Exposure**	A standardized Class I cavity should be prepared using a sterile high-speed bur under abundant sterile saline irrigation.<br>Pulp exposure should be achieved with a low-speed round bur under copious irrigation, creating a controlled exposure area of approximately 1 mm^2.<br>The cavity should be rinsed with sterile saline and gently dried with sterile cotton pellets.
**5. Biomaterial Application and Coronal Restoration**	The tested biomaterial should be placed in direct contact with the exposed pulp, typically in a 2–3 mm layer, and gently compacted using sterile cotton pellets.<br>Coronal sealing may be achieved by:<br>Filling the entire cavity with biomaterial, or<br>Covering the biomaterial with glass ionomer cement.<br>These variations do not appear to be the main determinant of biological outcomes, provided that an adequate seal and proper tooth suspension are ensured.

**6. Tooth Suspension and Culture Conditions**	Teeth should be fixed to an orthodontic wire and suspended in culture plates (commonly 24-well plates) so that the apex does not contact the bottom of the well. Each well should contain approximately 1.5 mL of DMEM supplemented with 10% FBS, antibiotics, and antifungal agents.<br>Incubation conditions: 37°C, 5% CO_2.<br>The culture medium should be replaced daily.
**7. Culture Duration**	A culture period of 3–4 weeks (21–28 days) is recommended to allow observation of early reparative dentinogenic events.<br>Available evidence supports cell survival and maintenance of mesenchymal stem cell characteristics for up to 4 weeks in this model.
**8. Histological Processing**	Following the culture period, specimens should undergo standard histological processing prior to analysis.
**9. Recommended Outcome Assessment**	Core histological staining:<br>Hematoxylin and Eosin (H&E)<br>Optional complementary analyses:<br>Immunohistochemistry markers: Nestin, Dentin sialoprotein (DSP), Collagen I, Osteonectin
